# Non-melanoma skin cancer treated with hypofractionated 192—Ir contact brachitherapy: a single institution series

**DOI:** 10.3389/fonc.2024.1525926

**Published:** 2025-01-17

**Authors:** Elisa Ciurlia, Bianca Santo, Maria Cristina Barba, Elisa Cavalera, Paola De Franco, Sara De Matteis, Giuseppe Di Paola, Angela Leone, Antonella Papaleo, Dino Rubini, Donatella Russo, Giuseppe Rubini, Angela Sardaro

**Affiliations:** ^1^ Radiation Therapy Unit, Department of Onco Hematology, ”Vito Fazzi” Hospital, Lecce, Italy; ^2^ Radiation Therapy Unit, Department of Precision Medicine, Università degli Studi della Campania Luigi Vanvitelli, Napoli, Italy; ^3^ Nuclear Medicine Unit, Interdisciplinary Department of Medicine, University of Bari, Bari, Italy

**Keywords:** non-melanoma skin carcinoma, basal cell carcinoma, squamous cell carcinoma, high-dose fractionated contact brachytherapy, personalization of treatment

## Abstract

**Background:**

Non-melanoma skin carcinoma (NMSC) is the most common malignant tumor in the population, with a steadily increasing incidence due to an aging population and sun exposure. The two main subtypes of NMSC are basal cell carcinoma(BCC) and squamous cell carcinoma(SCC). Therapeutic management of NMSC includes a variety of options, such as surgery, radiotherapy, and topical or systemic treatments. High-dose fractionated contact brachytherapy (c-HDR-BRT) is a viable therapeutic option for treating NMSCs.

**Methods:**

At our center, we treated 39 patients with BCC or SCC, with a total of 46 lesions, treated with c-HDR-BRT. The patients underwent two different radiotherapy schedules: 40 Gy in four fractions and 30 Gy in three fractions.

**Results:**

Two-year results showed 100% local control (LC) and 100% disease-specific survival (DSS), indicating high efficacy of c-HDR-BRT in terms of tumor control. Furthermore, the observed toxicity profile was favorable with no significant late toxicity.

**Conclusions:**

These results suggest that c-HDR-BRT represents a viable therapeutic alternative for NMSC, combining high oncological efficacy with an acceptable safety profile, while minimizing the aesthetic and functional impact of therapy. Finally, the study emphasizes the importance of personalization of treatment and careful evaluation of individual cases to optimize the treatment approach in NMSC.

## 1 Introduction

Non-melanoma skin cancers (NMSC) represent a highly prevalent condition that predominantly affects the elderly population. The increasing age of the general population, coupled with increased sun exposure, is the primary factor contributing to the clinical and economic burden associated with non-melanoma skin cancer. Primary therapeutic approaches include surgical excision, radiotherapy, and the administration of topical agents, each associated with distinct toxicity profiles. Hypofractionated contact high-dose rate brachytherapy (c-HDR-BRT) has emerged as a favorable treatment option, demonstrating advantages in terms of efficacy, reduced toxicity, and a positive socio-economic impact.

### 1.1 Epidemiology

Non-melanoma skin cancers, predominantly resulting from ultraviolet (UV) radiation exposure, are the most frequently diagnosed malignant tumors worldwide. More than 95% of these cancers occur in the head and neck region, including the nose, ears, eyelids, and lips. The two main types of NMSC are squamous cell carcinoma (SCC) and basal cell carcinoma (BCC) (1). A survey conducted in 2022 by the European Academy of Dermatology and Venereology (EADV) showed that approximately 7,304,000 Europeans were diagnosed with skin cancer, representing 1.71% of the adult European population (2). In Italy, BCC represents 15% of all neoplasms, with a standardized incidence rate of 31.9 cases per 100,000 inhabitants for males, and 22.8 cases per 100,000 for females. Focusing exclusively on invasive forms, SCC is the second most frequent skin cancer: its incidence is 4.2 cases per 100,000 inhabitants in males and 2.4 cases per 100,000 inhabitants in females (average incidence rate). It is noteworthy that approximately half of the patients affected by these conditions are over 65 years of age, and a significant proportion are likely to develop a second primary NMSC within five years of the initial diagnosis (3).

### 1.2 Etiopathogenesis

NMSC development is driven by a complex interplay between environmental and genetic factors. The primary environmental risk factor associated with NMSC is ultraviolet (UV) radiation from sunlight, particularly UVB rays, which are known to cause direct DNA damage UVB radiation inducing the formation of cyclobutane pyrimidine dimers (CPDs) and 6-4 photoproducts in the DNA structure, resulting in mutations if the damage is not adequately repaired by cellular mechanisms such as nucleotide excision repair (NER). A critical gene often mutated in NMSC is the p53 tumor suppressor gene, which plays a key role in controlling cell cycle arrest and apoptosis in response to DNA damage. Mutations in p53, especially those induced by UVB radiation (characterized by C→T transitions at dipyrimidine sites), are hallmark features of both BCC and SCC. Furthermore, genetic predisposition plays a significant role in the pathogenesis of NMSC (4). Conditions such as xeroderma pigmentosum (XP), which involves defects in the NER pathway, result in a diminished capacity to repair UV-induced DNA damage, leading to an elevated incidence of both BCC and SCC. Additionally, Basal Cell Nevus Syndrome (Gorlin syndrome), which arises from mutations in PTCH1, an integral component of the Hedgehog signaling pathway, also predisposes individuals to NMSC. These mutations result in the continuous activation of the pathway, leading to uncontrolled proliferation of basal cells and a predisposition to multiple BCCs. Immunosuppression is another significant factor contributing to the development of NMSC, particularly SCC (5). Immunosuppressive individuals exhibit a higher risk of developing NMSC owing to reduced immune surveillance. This allows for unchecked growth of abnormal cells that might otherwise be targeted by the immune system. Furthermore, Human Papillomavirus (HPV), particularly beta-HPV, has been implicated in the development of SCC in immunosuppressed individuals, primarily through its integration into the host genome, which disrupts the regulation of cell growth and apoptosis. At the molecular level (4, 6), the Hedgehog signaling pathway is critically involved in the pathogenesis of BCC. Mutations in PTCH1 or SMO genes lead to persistent activation of this pathway, promoting the proliferation of basal cells. The epidermal growth factor receptor (EGFR) pathway is often implicated in SCC, where overexpression or mutations in EGFR enhance cell proliferation, survival, and invasion, contributing to the invasive characteristics of SCC. Oxidative stress and chronic inflammation also play a role in the development of NMSC. Reactive oxygen species (ROS) produced by UV radiation and chemical carcinogens can cause oxidative DNA damage, resulting in mutations. Chronic inflammation, which often arises in contexts such as chronic wounds or scars, provides a microenvironment rich in proinflammatory cytokines and growth factors that can promote tumor initiation and progression (7).

### 1.3 Treatment options

Non-advanced NMSC typically has a more favorable prognosis than other malignancies. The primary treatment modalities include surgical intervention, radiotherapy, and topical agents applied directly to skin lesions (8–10).

Radiotherapy is generally recommended as the primary treatment option for patients who refuse surgery or are unsuitable for surgical procedures; it is also indicated for tumors located in areas where surgical excision may result in suboptimal cosmetic or functional outcomes. In cases with positive surgical margins or a high risk of recurrence, radiotherapy can also be recommended as adjuvant therapy. Additionally, alternative treatments, such as HDR-BRT (which is a form of interventional radiotherapy) offer promising solutions (11, 12). Recent advancements in technology and the commercial production of surface applicators have significantly improved the delivery of contact HDR-BRT for the treatment of NMSC. Contact HDR-BRT using radionuclide Iridium-192 (Ir-192). During contact HDR-BRT, a specially designed cup-shaped tungsten applicator containing the radiation source is placed in close contact with the tumor. Ir-192 emits gamma radiation at an energy level of 360 keV, which makes it a suitable source for brachytherapy targeting small superficial skin tumors measuring less than 5 mm in thickness (13). Although several studies have investigated the efficacy and toxicity of HDR-BRT, the main evidence is based on retrospective analysis using different techniques, doses, and fractionations (14). In this study, we report our monoinstitutional experience at the Radiotherapy Unit of “Vito Fazzi” Hospital in Lecce, Italy, in the treatment of BCC and SCC with hypofractionated c-HDR-BRT, with emphasis on efficacy and safety outcomes.

## 2 Materials and methods

This analysis encompasses patients with NMSC treated with contact high-dose-rate brachytherapy (c-HDR-BRT) at the Radiotherapy Department of “Vito Fazzi” Hospital (Lecce, Italy) between January 2017 and June 2024. All patients were evaluated by a multidisciplinary board that included dermatologists, plastic surgeons, oncologists, radiation oncologists, and pathologists. Histopathological confirmation of skin cancer was obtained for all the patients included in the study. Although optimal staging guidelines remain elusive, additional staging examinations (such as ultrasound or computed tomography (CT) scans) were performed based on the initial disease site and its drainage pathways, particularly in cases identified as high-risk for NMSC, according to clinical practice in brachytherapy (15, 16).

High-risk features are traditionally characterized by recurrent tumors, patient immunosuppression, lymphovascular or perineural invasion, poorly differentiated histology, lesions located on the ear or lip, tumor diameter >20 mm, and thickness exceeding 4 mm for SCC or diameter greater than 10 mm for BCC (17, 18).

**Figure 1:**
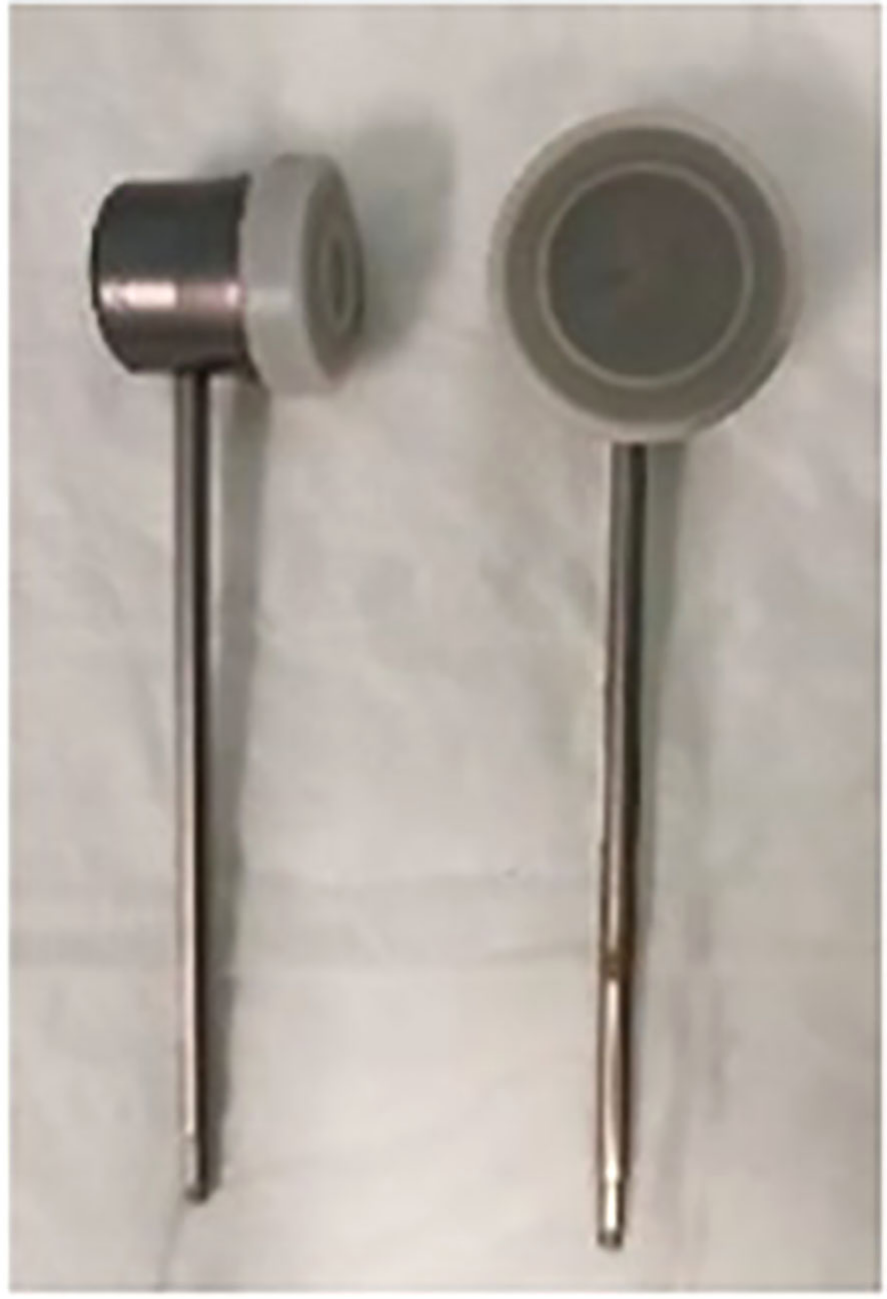
Leipzig applicators.

For locally advanced lesions suspected to extend into the bone or cartilaginous structures, computed tomography (CT) or magnetic resonance imaging (MRI) scans were requested. Patients with radiologically confirmed T4 disease, deeply ulcerated lesions or profound dermal infiltration were excluded from the study. In cases of larger lesions covered by eschar, topical galenic treatment (containing Vaseline, collagenase, and chloramphenicol) was applied daily for two weeks. Patients who exhibited healing of the eschar and reduction in lesion thickness were deemed for c-HDR-BRT treatment. Brachytherapy was administered as a definitive treatment in cases where critical health conditions contraindicated surgery, when surgery could potentially cause poor cosmetic or functional results, or when patients declined resection procedures. During the multidisciplinary board examination, gross tumor volume (GTV) was identified; a margin of 5 mm–10 mm for BCC and 10 mm–20 mm for SCC was added to define the clinical target volume (CTV). In cases of positive or narrow resection margins (less than 1 mm), after a multidisciplinary evaluation excluding the possibility of a second surgery for clear margins, c-HDR-BRT was administered as adjuvant treatment. CTV was defined as the skin area that included the surgical scar along with safety margins (5 mm–10 mm for BCC and 10 mm–20 mm for SCC). Depending on the extension of the CTV on the skin, Leipzig applicators of 10 mm (H1), 20 mm (H2), or 30 mm (H3) were utilized (**Figure 1**). During each treatment session, the patient was immobilized using an adjustable arm and an adhesive material. Two different doses and fractionation regimens were adopted: 40 Gy in four fractions or 30 Gy in three fractions. The dose prescription point was situated 3 mm–5 mm under the skin surface, based on the Groupe Européen de Curiethérapie (GEC) and European Society for Radiotherapy & Oncology (ESTRO) recommendations (19). Clinical outcomes and toxicity were recorded during follow-up visits and assessed by a team of three Radiation Oncologists. Acute and late toxicities were evaluated using the RTOG (Radiation Therapy Oncology Group) scale (20). Local control (LC), overall survival (OS), and disease-specific survival (DSS) were calculated using the Kaplan–Meier method.

## 3 Results

**Figure 2:**
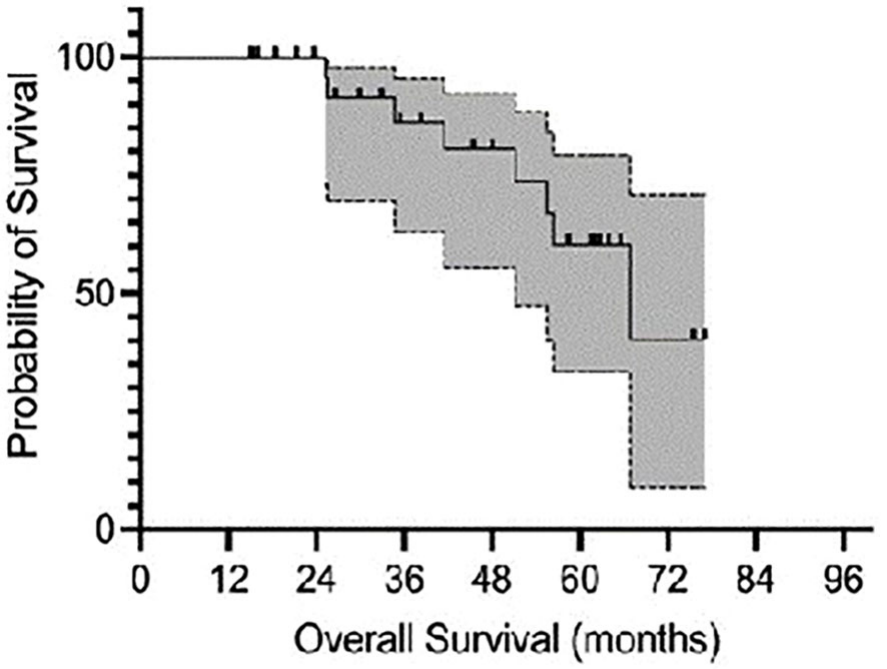
Kaplan–Meier curve representing OS.

**Figure 3:**
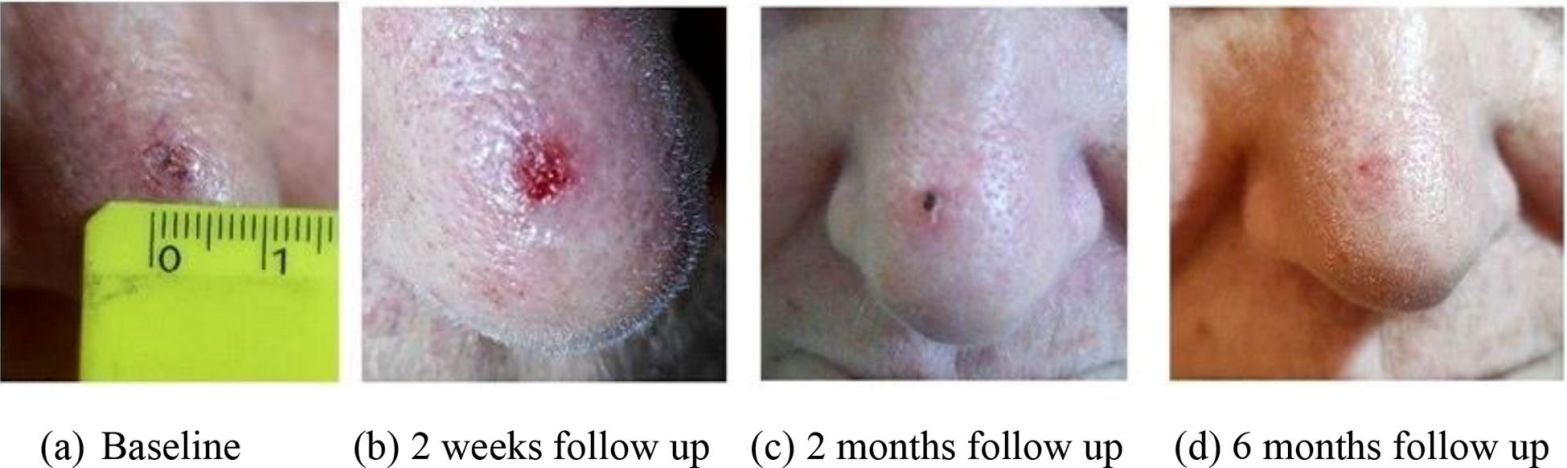
SCC of the nose treated with H2 applicator 40 Gy in four fractions. The image shows the lesion at baseline before brachytherapy **(A)**, two weeks after the end of treatment **(B)** and at 2 **(C)** and 6 **(D)** months of follow-up.

A total of 46 lesions from 39 consecutive patients (27 men and 12 women) with a median age of 80 years (range, 56 years–95 years) and a diagnosis of skin cancer were included in the study. The patients were treated with c-HDR-BRT between January 2017 and June 2024. Histopathological examination of the skin was performed in all patients: SCC in 17 patients (20 lesions, 37%) and BCC in 22 patients (26 lesions, 63%). The lesions were categorized based on their risk of recurrence: 61% were located in high-risk areas (central face, eyelids, eyebrows, periorbital area, nose, lips, chin, mandible, preauricular and postauricular regions, temples, ears, genitalia, hands, and feet); 37% were located in middle-risk areas (cheeks, forehead, neck, scalp), and 2% were found in low-risk areas (extremities). The additional details are provided in **Table 1**. Brachytherapy was delivered as definitive treatment for 25 lesions (54.3%) and as adjuvant treatment for 21 lesions (45.7%). The mean time from surgery to c-HDR-BRT was 3 months. The applicator size was: H1 in one lesion (2.2%), H2 in 12 lesions (26.1%) and H3 in 33 lesions (71.7%); the mean treatment delivery time was 474.5 s (7.9 min). Two different doses and fractionation regimens were adopted: 40 Gy in four fractions or 30 Gy in three fractions (**Table 2**). The mean follow-up period was 25.1 months (range: 1 month–77 months). The 2-year local control (LC), overall survival (OS), and disease-specific survival (DSS) rates were all recorded at 100%. It is noteworthy that seven patients died from unrelated causes (**Figure 2**). No acute toxicity was observed in 34.8% of cases. At the end of the treatment, RTOG acute toxicity classifications were Grade 1 (erythema, alopecia) in 39.1% of patients, Grade 2 (erythema and desquamation) in 10.9%, and Grade 3 (epitheliolysis and edema) in 15.2%. Late toxicity was not detected in 76.1% of the patients; the predominant late toxicity observed was hypopigmentation, classified as Grade 1, occurring in 21.7% of the cases. Grade 2 atrophy and telangiectasia were noted in 2.2% of the patients, with no incidents of Grade 3 late toxicity recorded (**Figure 3**).

**Table 1:** Patients and lesion characteristic.

Age (years)	Number (%)
Gender	
Male	27 (69.2%)
Female	12 (30.8 %)
Histology	
BCC	26 (63%)
SCC	20 (37%)
Lesion sites	
HIGH RISK	28 (61%)
Nose	20
Ear	6
Lip	1
Periocular	1
MIDDLE RISK	17 (37%)
Sculp	4
Neck	1
Cheech	1
Forehead	7
Zigomatic	4
LOW risk	1 (2%)
Leg	1

**Table 2:** Treatment characteristic.

Dose and fractionation	
40 Gy/4 fx	23
30 Gy/3 fx	23
Liepzig applicator size	
H1 2.2%	
H2 26.1%	
H3 71.7%	
Treatment delivery time 474.5 s	
RTOG Acute Toxicity	
G0 34.8%	
G1 39.1% (erytema, alopecia)	
G2 10.9% (erytema, desquamation)	
G3 15.2% (epitheliolysis and edema)	
RTOG Late Toxicity	
G0 76.1%	
G1 21.7% (ipopigmentation)	
G2 2.2% (atrofia and telengectasia)	
G3 0%	

## 4 Discussion

Contact HDR-BRT has become a pivotal treatment modality for cutaneous cancers and is characterized by its precision and effectiveness. The Leipzig applicator was designed with a concave structure that allows easy placement on the skin surface, ensuring a uniform dose distribution. The concave shape enables the applicator to closely conform to the body’s curvature and maintain tight contact between the radiation source and target lesion. The Valencia applicator is another advanced device used in c-HDR-BRT, characterized by its cylindrical geometry. Over the years, this field has witnessed significant advancements in techniques and technologies. Numerous studies have been conducted on c-HDR-BRT using both the Leipzig and Valencia applicators. The first and most comprehensive experience was reported in 1999 by Köhler-Brock et al., who treated 520 lesions using the Leipzig applicator, and a treatment regimen consisting of four to eight fractions, each delivering 5 Gy to 10 Gy. Following a long follow-up period of 10 years, the study reported a local control rate of 91% (21).

Another significant study conducted by Gauden et al. in 2013 (22) examined 236 lesions treated with 12 fractions of 3 Gy each. After a follow-up duration of 66 months, a local control rate of 98% was observed, with 71% of the patients experiencing Grade 1 toxicity and 34% experiencing Grade 2 toxicities. Similar results were reported in 2014 when Tormo (23) and colleagues explored the use of the Valencia applicator on 45 lesions, using a regimen of six to seven fractions of 6 Gy to 7 Gy. This study reported a remarkable local control rate of 98% with no significant toxicity noted after a follow-up of 47 months Delishaj et al. (24) expanded the application of HDR brachytherapy in 2015 to encompass a broader range of histologies, treating 57 lesions with the Valencia applicator in a regimen of eight to 10 fractions of 5 Gy each. This study achieved a local control rate of 96%, with minimal acute and late toxicity reported. In 2021, Pellizon et al. (25) evaluated 101 lesions treated with the Leipzig applicator using a regimen of seven to 22 fractions of 2.5 Gy–6 Gy. The study reported a local control rate of 92.1% with a few instances of Grade 3 toxicity. In the same year, Taylor (26) and colleagues focused on a smaller cohort, treating 20 lesions with six to seven fractions of 6 Gy to 7 Gy, also using the Leipzig applicator. They observed a local control rate of 94.7% and provided detailed reports on cutaneous toxicities, with assessments at baseline and 2 weeks, 3 months, and 6 months following treatment. They reported that 85% of the patients experienced acute Grade 2 toxicity.

Finally, Laliscia’s (27) study investigated 182 lesions treated with the Valencia applicator, with a treatment protocol of eight fractions of 5 Gy. After a median follow-up of 14 months, the study reported a local control rate of 90% accompanied by mild acute toxicity and minimal late toxicity.

Collectively, these studies demonstrated that c-HDR-BRT is a highly effective option for treating NMSC, offering high local control rates in conjunction with generally manageable toxicity profiles (**Table 3**). We report the experience of a single Radiotherapy Institution conducted on 39 NMSC patients (46 lesions) assessed by a multidisciplinary team and treated with the Liepzig applicator HDR-BRT. Most lesions (61%) were located on the face, specifically in high-risk areas such as the nose, ears, lips, and periorbital skin. The treatment regimen was selected from two hypofractionated schedules: 40 Gy in four fractions or 30 Gy in three fractions.

**Table 3:** Summary of studies on HDR brachytherapy with contact applicator.

Author	Year	Histology	N. lesions N. patients	Macroscopic Lesion/microscopic margins	Applicator	N. fx	Dose/fx	Frequency	Total Dose	Follow up (median)	Local control	Acute toxicity	Late toxicity
Köhler-Brock (21)	1999	BCC SCC Kaposi’s sarcoma Lymphomas Melanomas	520 lesions 520 patients	NA	Leipzig	4–8	5 Gy–10 Gy	1–2 times a week	30 Gy–40 Gy	10 years	91%	NA	NA
Gauden (22)	2013	BCC (121) SCC (115)	236 lesions 200 patients	162 macroscopic 74 positive margins	Leipzig	12	3 Gy	daily	36 Gy	66 months	98%	G1 71% G2 34%	5.5% hypopigmentation
Tormo (23)	2014	BCC (45)	45 lesions 32 patients	45 macroscopic	Valencia	6–7	6 Gy–7 Gy	Twice a week	42 Gy	47 months	98%	≥G2 0%	≥G2 0%
Delishaj (24)	2015	BCC (44) SCC (12) Kaposi’s Sarcoma (1)	57 lesions 39 patients	45 macroscopic 12 positive margins	Valencia	8–10	5 Gy	2–3 times a week	40 Gy–50 Gy	12 months	96%	G1 58% G2 5.3 %	G1 17% G2 1.9 %
Pellizon (25)	2021	BCC (70) SCC (31)	101 lesions 71 patients	101 macroscopic	Leipzig	7–22	2.5 Gy–6 Gy	3–5 times a week	40–55 Gy	42.8 months	92.1%	G3 8.9 %	G3 3.9%
Taylor (26)	2021	BCC (16) SCC (3)	20 lesions 19 patients	20 macroscopic	Leipzig	6–7	6–7	2–3 times a week	42 Gy	7.2 months	94.7%	G1 15% G2 85%	G0 100%
Laliscia (27)	2021	BCC (112) SCC (70)	182 lesions 95 patients	169 macroscopic 13 positive margins	Valencia	8	5	2–3 times a week	40 Gy	14 months	90%	G1 27% G2 6%	G1 36.7% G2 0.5 %

NA, Not available data.

To our knowledge, this represents the first experience of a Leipzig applicator utilizing c-HDR-BRT to deliver a dose of 10 Gy per fraction on consecutive days. Because of the limitations of the linear-quadratic model, which is not applicable for doses exceeding 7 Gy–8 Gy per fraction (28, 29), it is incorrect to conduct a dosimetric comparison between this study and other study series (21–24) in terms of calculated Biological Effective Doses (BEDs) and Equivalent Dose (EQD2) (setting alpha/beta ratio for skin cancer 10). Therefore, the following analyses provide descriptive comparisons of the different experiences available in the literature.

The safety profile recorded in this study appears to be similar to that of a previously published series (21–27). The incidence of acute RTOG toxicity G2 was 10.9% and G3 was 15.2%; the worst late toxicity was detected in one patient who experienced G2 atrophy and telangiectasia (2.2%). This toxicity profile demonstrates that contact HDR-BRT may represent an optimal technique for hypofractionation, owing to its technical and dosimetric advantages. Applicators ensure rapid lateral dose fall-off, preserving the surrounding healthy tissues and low penetration capacity of the radioactive source, sparing the underlying dermal and cartilaginous structures. With a mean follow-up time of 25.1 months, both local control and disease-specific survival rates were 100%. Excellent results were achieved in two studies, which reported a local control rate of 98% with a total dose of 36 Gy (3 Gy per fraction) (22) and 42 Gy (6 Gy–7 Gy per fraction) (23).

It appears reasonable to investigate the slight efficacy differences in the radiobiological, microenviromental, and immunological responses to hypofractionation.

Delivering high radiation doses directly to the tumor generates irreparable DNA damage, especially double-strand breaks. At a radiation dose of 10 Gy, the density of DNA damage increases, surpassing the capacity of cellular repair mechanisms such as non-homologous end joining and homologous recombination. The presence of DNA complex lesions, which pose considerable challenges for cellular repair, results in high rates of apoptosis and mitotic catastrophe. Rapid dose delivery in HDR-BRT exacerbates this effect, contributing to enhanced tumor control (30, 31).

Hypofractionation, defined as the administration of doses per fraction higher than 7 Gy–8 Gy, significantly influences the tumor microenvironment by inducing vascular damage and altering immune responses (32). The release of tumor-associated antigens and damage-associated molecular patterns contribute to a more robust immune-mediated tumor response. Understanding these aspects is crucial for optimizing treatment strategies and improving outcomes in cutaneous tumor management. A further and significant consideration deserves to be made regarding the median age (80 years) of the study population, which seems to be effective and safe even for the elderly, particularly among frail patients who may be unfit for surgery or longer RT schedules. From a clinical perspective, a schedule comprising three to four consecutive fractions appears to be highly advantageous in terms of compliance among elderly patients and their caregivers. This approach also assists in containing the economic burden associated with a prevalent disease, which significantly affects healthcare costs (33).

This study has some limitations that need to be highlighted. First and foremost, this is a retrospective analysis; this limitation could be mildly mitigated by the implementation of a uniform and standardized methodology: all patients were assessed by a multidisciplinary team, including dermatologists, plastic surgeons, oncologists, radiation oncologists, and pathologists; they were administered the same dose per fraction across two treatment schedules, and follow-up was conducted by the same group of three radiation oncologists. The short duration of follow-up and small sample size are also significant limitations, although these can be overcome with time and additional data. Additionally, the inclusion of both operated and non-operated patients in the same cohort introduces a significant bias in the assessment of efficacy outcomes; nevertheless, at the time of writing, no other available studies have reported c-HDR-BRT experience in an exclusive adjuvant setting. Data regarding postoperative adjuvant HDR-BRT in NMSC are limited and usually have to be extrapolated from NMSC series that also include treatments with definitive intent (22, 24, 27).

Despite these limitations, our study reports the efficacy and safety of hypofractionated c-HDR-BRT using a Leipzig applicator in an elderly population managed by a multidisciplinary team.

## 5 Conclusion

This study aimed to present the outcomes of our mono-institutional series using c-HDR-BRT for NMSC, employing the Leipzig applicator and two hypofractionated treatment schedules, each delivering 10 Gy per fraction. The results showed that c-HDR-BRT is an effective and safe treatment option for NMSC, suitable for facial lesions and elderly patients. Although robust and comparable scientific evidence is lacking, recommendations, together with multidisciplinary management, represent the key to ensuring the best therapeutic strategies. Further clinical trials are needed to optimize the selection criteria for this treatment modality, the treatment regimens, and to investigate the potential integration of immunotherapy and target agents.

## Data Availability

The original contributions presented in the study are included in the article/supplementary material. Further inquiries can be directed to the corresponding author.

## References

[1] Cell S. Skin Cancer: Basal and Squamous Cell What are the key statistics about basal and squamous What are basal and squamous cell skin cancers. (2013), 1–6. Available at: https://www.cancer.org/cancer/types/basal-and-squamous-cell-skin-cancer/about/key-statistics.html.

[2] Trakatelli M Richard MA Rouillard A Paul C Röcken M Stratigos A. The burden of skin disease in Europe. J Eur Acad Dermatol Venereol. (2023) 37:3–5. doi: 10.1111/jdv.19390 37806000

[3] AIRTUM Working Group Busco S Buzzoni C Mallone S Trama A Castaing M. Italian cancer figures–Report 2015: The burden of rare cancers in Italy. Epidemiol Prev. (2016) 40:1–120. doi: 10.19191/EP16.1S2.P001.035 26951748

[4] D’Orazio J Jarrett S Amaro-Ortiz A Scott T. UV radiation and the skin. Int J Mol Sci. (2013) 14:12222–48. doi: 10.3390/ijms140612222 PMC3709783 23749111

[5] Ramsay HM Fryer AA Hawley CM Smith AG Nicol DL Harden PN. Epidemiology and Health Services Research Non-melanoma skin cancer risk in the Queensland renal transplant population. Br J Dermatol. (2002) 147(5):950–6. doi: 10.1046/j.1365-2133.2002.04976.x 12410706

[6] Boukamp P. Non-melanoma skin cancer: What drives tumor development and progression? Carcinogenesis. (2005) 26:1657–67. doi: 10.1093/carcin/bgi123 15905207

[7] Garbe C Leiter U. Epidemiology of melanoma and nonmelanoma skin cancer-the role of sunlight. Adv Exp Med Biol. (2008) 624:89–103. doi: 10.1007/978-0-387-77574-6_8 18348450

[8] Kauvar ANB Cronin T Roenigk R Hruza G Bennett R. Consensus for nonmelanoma skin cancer treatment: Basal cell carcinoma, including a cost analysis of treatment methods. Dermatologic Surg. (2015) 41:550–71. doi: 10.1097/DSS.0000000000000296 25868035

[9] Arielle AN Arpey CJ Hruza G Olbricht SM Bennett R. Consensus for nonmelanoma skin cancer treatment, part II: squamous cell carcinoma, including a cost analysis of treatment methods. Dermatologic Surg. (2015) 41:1214–40. doi: 10.1097/DSS.0000000000000478 26445288

[10] Thomson J Hogan S Leonardi-Bee J Williams HC Bath-Hextall FJ. Interventions for basal cell carcinoma of the skin. Cochrane Database Syst Rev. (2020) 2020. doi: 10.1002/14651858.CD003412.pub3 PMC8164471 33202063

[11] Peris K Fargnoli MC Kaufmann R Arenberger P Bastholt L Seguin NB. European consensus-based interdisciplinary guideline for diagnosis and treatment of basal cell carcinoma—update 2023. Eur J Cancer. (2023) 192. doi: 10.1016/j.ejca.2023.113254 37604067

[12] Stratigos A Garbe C Lebbe C Malvehy J Del Marmol V Pehamberger H. Diagnosis and treatment of invasive squamous cell carcinoma of the skin: European consensus-based interdisciplinary guideline. Eur J Cancer. (2015) 51:1989–2007. doi: 10.1016/j.ejca.2015.06.110 26219687

[13] Granero D Candela-Juan C Vijande J Ballester F Perez-Calatayud J Jacob D. Technical Note: Dosimetry of Leipzig and Valencia applicators without the plastic cap. Med Phys. (2016) 43:2087–90. doi: 10.1118/1.4944784 27147321

[14] Benkhaled S Van Gestel D Gomes da Silveira Cauduro C Palumbo S Marmol Vd Desmet A. The state of the art of radiotherapy for non-melanoma skin cancer: A review of the literature. Front Med. (2022) 9:913269. doi: 10.3389/fmed.2022.913269 PMC9272768 35833108

[15] Fox M Brown M Golda N Goldberg D Miller C Pugliano-Mauro M. Nodal staging of high-risk cutaneous squamous cell carcinoma. J Am Acad Dermatol. (2019) 81:548–57. doi: 10.1016/j.jaad.2018.09.006 PMC8081061 30227190

[16] Ouhib Z Kasper M Perez Calatayud J Rodriguez S Bhatnagar A Pai S. Aspects of dosimetry and clinical practice of skin brachytherapy: The American Brachytherapy Society working group report. Brachytherapy. (2015) 14:840–58. doi: 10.1016/j.brachy.2015.06.005 26319367

[17] Brantsch KD Meisner C Schönfisch B Trilling B Wehner-Caroli J Röcken M. Analysis of risk factors determining prognosis of cutaneous squamous-cell carcinoma: a prospective study. Lancet Oncol. (2008) 9:713–20. doi: 10.1016/S1470-2045(08)70178-5 18617440

[18] Mullen JT Feng L Xing Y Mansfield PF Gershenwald JE Lee JE. Invasive squamous cell carcinoma of the skin: Defining a high-risk group. Ann Surg Oncol. (2006) 13:902–9. doi: 10.1245/ASO.2006.07.022 16788750

[19] Guinot JL Rembielak A Perez-Calatayud J Rodríguez-Villalba S Skowronek J Tagliaferri L. GEC-ESTRO ACROP recommendations in skin brachytherapy. Radiother Oncol. (2018) 126:377–85. doi: 10.1016/j.radonc.2018.01.013 29455924

[20] Cox JD Stetz JA Pajak TF. Toxicity criteria of the Radiation Therapy Oncology Group (RTOG) and the European organization for research and treatment of cancer (EORTC). Int J Radiat Oncol Biol Phys. (1995) 31:1341–6. doi: 10.1016/0360-3016(95)00060-C 7713792

[21] Köhler-Brock A Prager W Pohlmann S KS. The indications for and results of HDR afterloading therapy in diseases of the skin and mucosa with standardized surface applicators (the Leipzig applicator). Strahlentherapie und Onkol. (1999) 175:347. doi: 10.1007/bf03039594 10230459

[22] Gauden R Pracy M Avery AM Hodgetts I Gauden S. HDR brachytherapy for superficial non-melanoma skin cancers. J Med Imaging Radiat Oncol. (2013) 57:212–7. doi: 10.1111/j.1754-9485.2012.02466.x 23551783

[23] Tormo A Celada F Rodriguez S Botella R Ballesta A Kasper M. Non-melanoma skin cancer treated with HDR valencia applicator: Clinical outcomes. J Contemp Brachytherapy. (2014) 6:167–72. doi: 10.5114/jcb.2014.43247 PMC4105643 25097557

[24] Delishaj D Laliscia C Manfredi B Ursino S Pasqualetti F Lombardo E. Non-melanoma skin cancer treated with high-doserate brachytherapy and Valencia applicator in elderly patients: A retrospective case series. J Contemp Brachytherapy. (2015) 7:437–44. doi: 10.5114/jcb.2015.55746 PMC4716125 26816500

[25] Pellizzon ACA Fogaroli R Chen MJ Maia P Gondim G de Castro Guedes D. High-dose-rate brachytherapy using leipzig applicators for non-melanoma localized skin cancer. J Contemp Brachytherapy. (2020) 12:435–40. doi: 10.5114/jcb.2020.100376 PMC7701921 33299432

[26] Taylor JM Dasgeb B Liem S Ali A Harrison A Finkelstein M. High-dose-rate brachytherapy for the treatment of basal and squamous cell carcinomas on sensitive areas of the face: A report of clinical outcomes and acute and subacute toxicities. Adv Radiat Oncol. (2021) 6:100616. doi: 10.1016/j.adro.2020.10.028 33912732 PMC8071728

[27] Laliscia C Coccia N Fuentes T Perrone F Paiar F. Two different sizes of Valencia applicators in non-melanoma skin cancer treatment with iridium-192 high-dose-rate brachytherapy. J Contemp Brachytherapy. (2021) 13:615–9. doi: 10.5114/jcb.2021.112111 PMC8782077 35079246

[28] Kirkpatrick JP Meyer JJ Marks LB. The linear-quadratic model is inappropriate to model high dose per fraction effects in radiosurgery. Semin Radiat Oncol. (2008) 18:240–3. doi: 10.1016/j.semradonc.2008.04.005 18725110

[29] Park C Papiez L Zhang S Story M Timmerman RD. Universal survival curve and single fraction equivalent dose: useful tools in understanding potency of ablative radiotherapy. Int J Radiat Oncol Biol Phys. (2008) 70:847–52. doi: 10.1016/j.ijrobp.2007.10.059 18262098

[30] Williams J. Basic clinical radiobiology. Int J Radiat Biol. (2019) 95:797–7. doi: 10.1080/09553002.2019.1569781

[31] Bentzen SM DW. High single doses in radiotherapy: challenges and solutions. Radiother Oncol. (2006) 81:1–13.16971009

[32] Dewan MZ Galloway AE Kawashima N Dewyngaert JK Babb JS Formenti SC. Fractionated but not single dose radiotherapy induces an immune-mediated abscopal effect when combined with anti- CTLA-4 antibody. Clin Cancer Res. (2009) 17:211–24. doi: 10.1158/1078-0432.CCR-09-0265.Fractionated PMC2746048 19706802

[33] Faur CI Moldovan MA Văleanu M Rotar H Filip L Roman RC. The prevalence and treatment costs of non-melanoma skin cancer in cluj-napoca maxillofacial center. Med. (2023) 59:1–13. doi: 10.3390/medicina59020220 PMC9968035 36837422

